# Phenotyping Young GluA1 Deficient Mice – A Behavioral Characterization in a Genetic Loss-of-Function Model

**DOI:** 10.3389/fnbeh.2022.877094

**Published:** 2022-06-02

**Authors:** Maria Reiber, Helen Stirling, Rolf Sprengel, Peter Gass, Rupert Palme, Heidrun Potschka

**Affiliations:** ^1^Institute of Pharmacology, Toxicology, and Pharmacy, Ludwig Maximilian University of Munich, Munich, Germany; ^2^Max Planck Institute for Medical Research, Heidelberg, Germany; ^3^RG Animal Models in Psychiatry, Department of Psychiatry and Psychotherapy, Central Institute of Mental Health, Medical Faculty Mannheim, Heidelberg University, Mannheim, Germany; ^4^Unit of Physiology, Pathophysiology and Experimental Endocrinology, Department of Biomedical Sciences, University of Veterinary Medicine Vienna, Vienna, Austria

**Keywords:** 3R, severity assessment, genetic mouse model, *GRIA1*, glutamate, schizophrenia, knockout, glucocorticoids

## Abstract

Alterations of glutamatergic neurotransmission have been implicated in neurodevelopmental and neuropsychiatric disorders. Mice lacking the GluA1 AMPA receptor subunit, encoded by the *Gria1* gene, display multiple phenotypical features associated with glutamatergic dysfunction. While the phenotype of adult GluA1 deficient (*Gria1^–/–^*) mice has been studied comprehensively, there are relevant gaps in knowledge about the course and the onset of behavioral alterations in the *Gria1* knockout mouse model during post-weaning development. Based on former investigations in young wild-type mice, we exposed female and male adolescent *Gria1^–/–^* mice to a behavioral home-cage based testing battery designed for the purpose of severity assessment. Data obtained from mice with a constitutive loss of GluA1 were compared with those from wild-type littermates. We identified several genotype-dependent behavioral alterations in young *Gria1^–/–^* mice. While the preference for sweetness was not affected by genotype during adolescence, *Gria1^–/–^* mice displayed limited burrowing performance, and reached lower nest complexity scores. Analysis of home-cage based voluntary wheel running performance failed to confirm genotype-dependent differences. In contrast, when exposed to the open field test, *Gria1^–/–^* mice showed pronounced hyperlocomotion in early and late adolescence, and female *Gria1*^–/–^ mice exhibited thigmotaxis when prepubescent. We found increased corticosterone metabolite levels in fecal samples of adolescent *Gria1^–/–^* mice with females exhibiting increased adrenocortical activity already in prepubescence. Considering the course of behavioral modifications in early and late adolescence, the results do not support a persistent level of distress associated with GluA1 deficiency in the line. In contrast, the laboratory-specific readouts indicate transient, mild impairments of behavioral patterns relevant to animal welfare, and suggest a mild overall burden of the line.

## Introduction

There is compelling evidence that alterations of glutamatergic neurotransmission are implicated in neuropsychiatric disorders (Sanacora et al., 2012; Uno and Coyle, 2019). In particular, the incorporation of the L-alpha-amino-3-hydroxy-5-methylisoxazole-4-propionate (AMPA) glutamate receptor subunit 1 (GluA1) into synaptic AMPA receptors is considered to be a key mechanism of synaptic structural as well as functional plasticity (Sprengel et al., 1998; Hayashi et al., 2000; Shi et al., 2001; Malinow and Malenka, 2002; Kopec et al., 2007; Lee et al., 2010; Huganir and Nicoll, 2013). In humans the AMPA receptor subunit GluA1 is encoded by the *GRIA1* gene, a locus with genome-wide association to schizophrenia (Ripke et al., 2013; Schizophrenia Working Group of the Psychiatric Genomics Consortium, 2014). *Gria1* deficient mice, harboring the global loss of GluA1, mimic multiple features of neuropsychiatric disease states related to glutamatergic dysfunction, among others, schizophrenia and schizoaffective disorders, attention deficit hyperactivity disorders (ADHD), bipolar disorders, and mood disorders (Wiedholz et al., 2008; Fitzgerald et al., 2010; Ben Abdallah et al., 2011; Barkus et al., 2012; Sanacora et al., 2012; Vogt et al., 2014). Data from adult mice suggest that the global loss of GluA1 induces a complex behavioral phenotype, comprising spatial working memory deficits with an intact reference memory (Zamanillo et al., 1999; Reisel et al., 2002; Schmitt et al., 2003; Bannerman et al., 2004), novelty-induced hyperexcitability (Wiedholz et al., 2008) and hyperlocomotion (Vekovischeva et al., 2001; Schmitt et al., 2003; Wiedholz et al., 2008), as well as a depression-associated phenotype (Chourbaji et al., 2008; Austen et al., 2017). While the behavioral phenotype of adult GluA1 deficient mice has been studied extensively, there is still limited information on the course and the onset of behavioral modifications during post-weaning development. Here, we aimed to profile aspects of the behavioral phenotype of young *Gria1* knockout mice as a genetic loss-of-function model, exposing female and male *Gria1*^–/–^ mice to a home-cage based testing battery during post-weaning development. The applied behavioral and biochemical composite measure scheme was derived from an extensive dataset of consortium data for the purpose of severity assessment in different adult rodent models (Bleich et al., 2020). Our group has further figured out the parameters rendering the highest informative value based on a bioinformatic approach (Keubler et al., 2020; van Dijk et al., 2020), and we subsequently have evaluated their versatile applicability in adolescent C57BL/6J wild-type mice (Reiber et al., 2022). Despite the aspect of ethical responsibility that researchers bear, animal welfare assessments are mandatory according to Directive 2010/63/EU. For implementation of the Directive 2010/63/EU, animal experiments have to be designed to reduce pain, suffering, distress or lasting harm to a minimum (Smith et al., 2018). Unfortunately, there are still relevant gaps in knowledge on evidence-based, validated methods to determine and grade levels of distress and to decipher the multidimensional aspects of life-time severity (Keubler et al., 2020). Particularly in neuroscience research, genetic mouse models can experience peaks of distress during the course of post-weaning development (Sukoff Rizzo and Crawley, 2017). GluA1 deficient mice of the strain B6N.129-Gria1^*TM* 1*Rsp*^ represent a loss-of-function model of neuropsychiatric relevance, which has been classified to experience “no harm burden.” However, following the guidelines provided by the German Centre for the Protection of Laboratory Animals (Bf3R), the entire developmental phase of the mice between weaning in late infancy and young adulthood is not necessarily involved when investigating severity grading of genetically modified lines (EU, 2010; Bf3R, 2016; Reiber et al., 2022). Ethologically-based characterization, comprising cross-model, cross-species and cross-age validations, allow for conclusions about face validity (Percie du Sert et al., 2020), adequate refinement measures and welfare-based model prioritization (van Dijk et al., 2020). Our findings aim to shed light on subtle levels of distress associated with genetic deficiency, here *Gria1^–/–^*, and provide further information for conclusions on cumulative severity grading and recommendations for refinement of genetic mouse lines.

## Materials and Methods

### Ethics Statement

All animal experiments were conducted and reported in line with the EU Directive 2010/63/EU, the German Animal Welfare Act, the ARRIVE (Animal Research: Reporting of *In Vivo* Experiments) guidelines, and the Basel declaration^1^ including the 3R principle. All animal experiments were approved by the government of Upper Bavaria (Munich, Germany, license number ROB-55.2-2532.Vet_02-19-157).

### Animals

*Gria1* knockout (*Gria1^–/–^)* mice (line B6N.129-*Gria*1^TM 1*Rsp*^/J, available at The Jackson Laboratory: Strain #019011, Mouse Genome Informatics ID: MGI:2178057) were generated as described previously (Zamanillo et al., 1999), and have been backcrossed into C57BL6/N background for more than 10 generations at the animal facility of Heidelberg University (IBF, Heidelberg). Experimental animals were bred at the Institute of Pharmacology, LMU Munich, from *Gria1* heterozygous (HET) x HET parents (19 HET females, 7 HET males), which were obtained from the IBF, Heidelberg. Offspring animals were divided into the experimental group carrying the genetic *Gria1* deficiency, referred to as “*Gria1^–/–^* mice” in the following (*n* = 18, female: male, *n* = 10:8), and the wild-type control group, referred to as “wild-type mice” in the following (*n* = 20, female: male, *n* = 10:10). Offspring animals were genotyped prior to weaning by ear punch biopsy as described previously (Zamanillo et al., 1999). The genotypes of experimental animals were confirmed by a second PCR after completion of the experiments.

Breeding animals were housed in Makrolon type III cages (Ehret GmbH & Co. KG, Emmendingen, Germany), enriched with bedding material (Lignocel Select, J. Rettenmaier & Söhne GmbH & Co. KG, Rosenberg, Germany), Enviro Dri nesting material (Enviro Dri, Claus GmbH, Limburgerhof, Germany), two nestlets (Ancare, Bellmore, NY, United States), and one square animal house (Zoonlab GmbH, Castrop-Rauxel, Germany).

From weaning at postnatal day (P) 21 onward, experimental animals were housed in groups of two, according to their *Gria1*-genotype and sex, as one experimental unit (*n* = 2). Experimental units (*n* = 19) were housed under controlled environmental conditions (22–24°C, 45--60% humidity) in a 12-h dark-light cycle with *ad libitum* access to food (Ssniff Spezialdiäten GmbH, Soest, Germany) and tap water, in Makrolon type III cages (Ehret GmbH & Co. KG, Emmendingen, Germany), provided with bedding material (Lignocel Select, J. Rettenmaier & Söhne GmbH & Co. KG, Rosenberg, Germany), two nestlets (Ancare, Bellmore, NY, United States) and one square animal house (Zoonlab GmbH, Castrop-Rauxel, Germany) per cage. The order of cages was randomized^2^. Animals received a fresh cage once per week. During 4 days each in the phases of prepubescence and sexual maturity, experimental units were housed in home-cage systems with continuous video recording (PhenoTyper, Noldus, Wageningen, Netherlands), combined with the video analysis tracking software EthoVision XT 15 (EthoVision XT, SCR_000441). Each PhenoTyper cage was supplemented with 200 g bedding material (Lignocel Select, J. Rettenmaier & Söhne GmbH & Co. KG, Rosenberg, Germany), two nestlets (Ancare, Bellmore, NY, United States), an infrared translucent shelter (Noldus, Wageningen, Netherlands) and two drinking bottles (Noldus, Wageningen, Netherlands).

### Experimental Design

**FIGURE 1:**
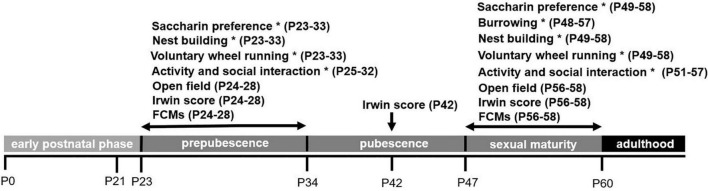
Overview of the experimental scheme. FCMs refers to fecal corticosterone metabolites, * refers to behavioral tests conducted in the animals’ home cages. Age ranges of test conduction [postnatal days (P)] are provided in the brackets.

Mice were investigated at different ages during the phase of murine adolescence, in line with a sub-classification of the adolescence phase as suggested by Brust et al. (2015): (1) prepubescence (P23 onward), (2) pubescence (P35 onward), and (3) sexual maturity (P48 onward). In addition, we assessed the condition of the litter/newborns based on the suggestions by the Bf3R (Bf3R, 2016). We use the term “sexual maturity” when referring to the period of late adolescence in which fully fertile animals start to disperse (Brust et al., 2015). Since several of the tests were conducted in the animals’ home cages, respective tests were analyzed per experimental unit (*n* = 2), as indicated in Figure 1. Moreover, age ranges of the animals (*n* = 38; experimental units: *n* = 19) passing the testing battery are illustrated in Figure 1, providing an overview of the experimental scheme.

### Behavioral Home-Cage Assessment

#### Saccharin Preference

The saccharin preference test was applied twice during adolescence, in prepubescent mice and once again when they had reached sexual maturity. The preference for sweetness can be interpreted to reflect anhedonia-associated behavioral traits in laboratory rodents (Crawley, 2006; Klein et al., 2015). As described previously (Reiber et al., 2022), mice received two water bottles filled with 200 g tap water on the first and third day during the two observation periods in order to assess the water intake over 24 h. On day 2 and 4, one water bottle was filled with 200 g of a 0.1% saccharin solution (Aldrich Saccharin ≥ 98%, Sigma-Aldrich Chemie GmbH, Taufkirchen, Germany). The other bottle contained 200 g of tap water. The side of the bottle containing the saccharin solution in the cage was alternated on day 2 and 4. We performed the analysis following a protocol by Klein and colleagues (Klein et al., 2015).

#### Burrowing

Since we have detected relevant levels of burrowing activity in adolescent wild-type mice once the animals reached sexual maturity (Reiber et al., 2022), we applied the burrowing test in sexually mature mice only. We analyzed the burrowing performance assessing the weight of food pellets burrowed by the animals in their home cages during a 2-h light-phase session and thereafter once again during the dark phase overnight. Overnight and light-phase burrowing performance were assessed in sexually mature mice on two consecutive days. An empty water bottle (length: 20 cm, diameter of the bottleneck: 3.5 cm; Zoonlab GmbH, Castrop-Rauxel, Germany) was filled with 200 ± 1 g food pellets (Ssniff Spezialdiäten GmbH, Soest, Germany) and placed into the home-cage approximately 2 h prior to the beginning of the dark phase. After exactly 2 h, the weight of the bottle with the remaining pellets was measured. Pellets distributed on the floor of the cage from the previous 2-h test session were removed. The bottle was then placed back in the cage for the assessment of overnight burrowing performance. Directly after the end of the dark phase on the next day, the weight of the bottle with the remaining pellets was measured.

#### Nest Building

As described previously (Reiber et al., 2022), we assessed nest building performance in the home-cage, analyzing the complexity and shape of the nest over an observation period of 4 days each in prepubescent and sexually mature mice. Mice received two pressed cotton squares per home cage. Pictures of the nest were taken on a daily basis in the morning, including a top-down view and two side views at an angle of 90 and approximately 45 degrees. The image-based evaluation of nest complexity was carried out using a slightly modified version of the protocol provided by Jirkof et al. (2013). The scoring of the images was carried out by a person unaware of group allocation. For further information on the applied scoring scheme, see the Supplementary File.

#### Voluntary Wheel Running and Bench-Top Assessment

Mice had free access to running wheels when prepubescent and once sexually mature over a period of 4 days each. We assessed voluntary wheel running performance in the animals’ familiar home-cage environment as described previously (Reiber et al., 2022). In short: the PhenoTyper cages were provided with a freely accessible running wheel (diameter: 15 cm, width: 7 cm; PhenoWheel, Noldus, Wageningen, Netherlands) for 24 h per day. Analysis was carried out using the software EthoVision XT 15 (EthoVision XT, RRID:SCR_000441), based on the counts (one rotation of the wheel) registered per minute.

Bench-top assessment (Baran et al., 2022) focusing on activity patterns was conducted in the PhenoTyper cages using the tracking software Ethovision XT 15. On the third day after the mice were introduced to the PhenoTyper cages (adaptation time approximately: 60–64 h), we analyzed parameters of activity (distance, velocity) during a time slot of exactly 60 min shortly after the beginning of the dark phase. We started the analysis approximately 30 min after the beginning of the dark phase, since animals showed high levels of activity then. In addition, the location of the mice in their home cages was tracked simultaneously. We analyzed the durations mice spent in three zones: (1) the area surrounding the feeder rack (zone “feeding”), (2) the area surrounding the drinking bottles (zone “drinking”), and (3) the center of the cage (zone “center”). We started the analysis approximately 30 min after the beginning of the dark phase, since animals showed high levels of activity then. The tracking duration lasted exactly 60 min. Analysis was performed during prepubescence and once again during sexual maturity. Parameters indicating features of social interaction displayed by the two animals per home-cage were analyzed simultaneously with the analysis of activity patterns. Hereby, the duration of body contact and the mean distance between the two subjects were analyzed using the software Ethovision XT 15.

#### Open Field

We applied the open field test assessing exploratory behavior and locomotor activity with a monitoring duration of 15 min. As described previously (Reiber et al., 2022), male animals were tested prior to female animals. Animals were placed individually in a white, circular shaped open field arena (diameter: 60 cm; lighting condition: 20 lux) 10 cm away from and facing the wall. The open field arenas were cleaned with 0.1% acetic acid after each trial. For analysis the arena was subdivided into the zone “wall,” comprising the outer 17% of the arena, and the zone “center,” comprising the inner 45% of the arena. Analysis was carried out using the tracking software EthoVision XT 8.5 (EthoVision XT, RRID:SCR_000441). The frequency of “rearing” positions and “jumps” against the arena wall were assessed manually by a person blinded for group allocation.

#### Irwin Score

As described previously (Reiber et al., 2022), we applied the traditional Irwin scoring system (Irwin, 1968) to assess the general condition of the mice and to obtain information about general behavioral, neurological, and vegetative changes. Irwin scoring of handling-associated parameters was carried out directly after the open field test between 07:00 a.m. and 01:00 p.m. The time interval per assessment per mouse was approximately 2 min. Irwin scoring was split into three consecutive parts, followed by rectal body temperature measurement. Irwin scoring was conducted by the same person for all assessments and the assessor was unaware of group allocation. For detailed information on the applied modified scoring system, see the Supplementary File.

#### Body Weight

We closely monitored the development of body weight from weaning onward during late infancy and adolescence on the following postnatal days: P21, P23, P25, P27, P30, P36, P42, P49, and P55.

#### Fecal Corticosterone Metabolites

We analyzed adrenocortical activity in prepubescent and sexually mature mice as described previously (Reiber et al., 2022). Individual fecal samples for the analysis of corticosterone metabolites were collected directly after the open field paradigm. For detailed information on collecting, processing, and biochemical analysis, see the Supplementary File.

#### Statistics

Statistical analysis was conducted using GraphPad Prism 5.04 for Windows (GraphPad Prism Software, San Diego, CA, United States) and R version 4.0.2 (R Core Team, 2020). Graphical illustration was conducted with GraphPad Prism 5.04. We analyzed group differences by parametric two-way analysis of variance (ANOVA) or non-parametric aligned rank transform (ART) ANOVA (Wobbrock et al., 2011; Elkin et al., 2021) using ARTool (version 2.1.2, Washington, DC, United States) with genotype and sex as factors. Main effects were further investigated applying false discovery rate (FDR) correction with the Benjamini–Hochberg method to adjust *p* values. In cases of a significant genotype by sex interaction, Bonferroni multiple comparison *post hoc* tests were applied. A *p*-value < 0.05 was considered statistically significant. For nest complexity scores, the median is shown. All other data are expressed as mean ± standard error of the mean (SEM).

## Results

### Behavioral Home Cage Assessment

#### Saccharin Preference

Analyses of saccharin preference did not reveal a significant effect of genotype in prepubescent and in sexually mature mice. Data from female and male mice did not differ in a significant manner and genotype by sex interactions were not significant (Supplementary Figure 1).

#### Burrowing

**FIGURE 2:**
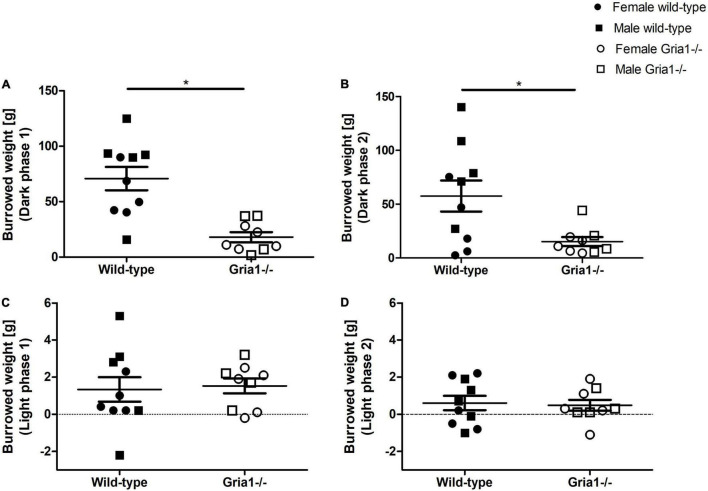
Burrowing performance. The assessment of burrowing revealed a significant effect of genotype on the amount of burrowed pellets in the first overnight test session **(A)** and in the second overnight session on the consecutive day **(B)**. The analysis of burrowing performance during the 2-h light session showed significant group differences neither in the first test session **(C)** nor in the second session on the consecutive day **(D)**. ANOVA, followed by FDR correction. *n* = 4–5 per genotype per sex. *p < 0.05. Error bars indicate the standard error of the mean (SEM).

The assessment of burrowing performance in sexually mature mice revealed a significant effect of genotype with *Gria1^–/–^* mice burrowing smaller amounts of pellets overnight than wild-type littermates in the first test session (Figure 2A, ANOVA: genotype *F*_1,15_ = 20.07, *p* = 0.0004, FDR-adjusted *p* = 0.003) and in the second test session on the consecutive day (Figure 2B, ANOVA: genotype *F*_1,15_ = 10.24, *p* = 0.006, FDR-adjusted *p* = 0.03). Analyses indicated no significant effects of sex and no significant genotype by sex interaction for both overnight sessions. The assessment of burrowing performance during the two 120-min light sessions demonstrated a low mean performance without significant group differences (Figures 2C,D).

#### Nest Building

**FIGURE 3:**
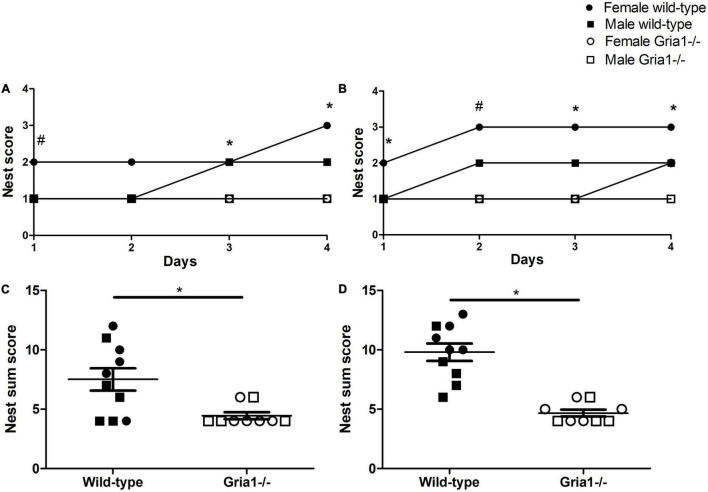
Nest building performance and nest complexity. The assessment of nest building performance during the 4-day observation period showed that in prepubescent **(A)** and sexually mature mice **(B)** mice, a significant effect of genotype on nest complexity scores was detected for several of the four observation days. Genotype significantly interacted with sex, reflecting decreased nest scores in female *Gria1^–/–^* mice when compared to female wild-type littermates on the first day in prepubescent mice **(A)** and on the second day in sexually mature mice **(B)**. The analysis of nest complexity sum scores revealed significantly decreased scores in prepubescent **(C)** and sexually mature **(D)** *Gria1^–/–^* mice as compared to wild-type littermates. ART ANOVA, followed by FDR correction or Bonferroni multiple comparison *post hoc* tests. *n* = 4–5 per genotype per sex. *p < 0.05 and #*p* < 0.05 (females). **(A,B)** Illustrate the median, in **(C,D)** error bars indicate the standard error of the mean (SEM).

In prepubescent mice, analyses of nest complexity scores confirmed a significant effect of genotype for each of the four observation days following the offer of new nesting material, except for the second day: Nests from *Gria1^–/–^* mice reached lower scores than those from wild-type littermates (Figure 3A, ART ANOVAs: day 1: genotype *F*_1,15_ = 18.04, *p* = 0.007; day 2: genotype *F*_1,15_ = 7.78, *p* = 0.01, FDR-adjusted *p* = 0.06; day 3: genotype *F*_1,15_ = 10.07, *p* = 0.006, FDR-adjusted *p* = 0.03; day 4: genotype *F*_1,15_ = 8.29, *p* = 0.01, FDR-adjusted *p* = 0.049). Significant effects of sex were absent, and there was no significant genotype by sex interaction, except for the assessment on the first observation day (Figure 3A, ART ANOVA: day 1: sex *F*_1,15_ = 5.86, *p* = 0.03, genotype by sex interaction *F*_1,15_ = 5.86, *p* = 0.03) indicating that on the first day nests from female *Gria1^–/–^* mice reached lower complexity scores than those from female wild-type littermates (Bonferroni *post hoc* test: *p* = 0.007).

Nest complexity scores in sexually mature mice were significantly affected by genotype on each of the four observation days reflecting that nests from *Gria1^–/–^* mice reached lower complexity scores than those from wild-types (Figure 3B, ART ANOVAs: day 1: genotype *F*_1,15_ = 18.62, *p* = 0.0006, FDR-adjusted *p* = 0.004; day 2: genotype *F*_1,15_ = 99.54, *p* < 0.0001; day 3: genotype *F*_1,15_ = 61.20, *p* < 0.0001, FDR-adjusted *p* < 0.0001; day 4: genotype *F*_1,15_ = 51.96, *p* < 0.0001, FDR-adjusted *p* < 0.0001). The analysis in sexually mature mice indicated a significant genotype by sex interaction on the second observation day (ART ANOVA: day 2: genotype by sex interaction *F*_1,15_ = 7.15, *p* = 0.02) with nests from female *Gria1^–/–^* mice reaching lower scores than those from female wild-type littermates (Bonferroni *post hoc* test: *p* = 0.0001). Independent of the genotype, sex effects on nest complexity scores from sexually mature mice were not significant.

Considering sum scores, calculated from the complexity scores of the individual days, there was a significant effect of genotype for the assessment during prepubescence (Figure 3C, ART ANOVA: genotype *F*_1,15_ = 10.19, *p* = 0.006, FDR-adjusted *p* = 0.029) and sexual maturity (Figure 3D, ART ANOVA, genotype *F*_1,15_ = 46.58, *p* < 0.0001, FDR-adjusted *p* < 0.0001), but genotype did not interact with sex in both cases. Sum complexity scores did not differ between sexes for both age phases.

#### Voluntary Wheel Running

**FIGURE 4:**
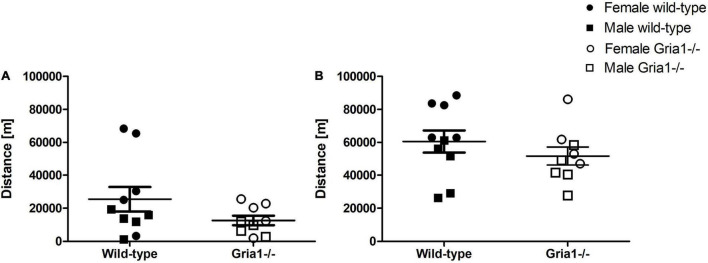
Voluntary wheel running. The analysis of voluntary wheel running performance, assessed in prepubescent **(A)** and again in sexually mature mice **(B)**, showed no significant effects of genotype on the total distance moved. ANOVA. *n* = 4–5 per genotype per sex. Error bars indicate the standard error of the mean (SEM).

Analyses of the distance moved in the running wheel failed to demonstrate a significant effect of genotype in prepubescent and sexually mature mice, and genotype by sex interactions were absent in both measurements (Figures 4A,B). Independent of genotype, there was a significant effect of sex on the distance run by sexually mature mice (ANOVA: sex *F*_1,15_ = 13.31, *p* = 0.002, FDR-adjusted *p* = 0.01) with females running greater distances than males.

#### Bench-Top Assessment

**FIGURE 5:**
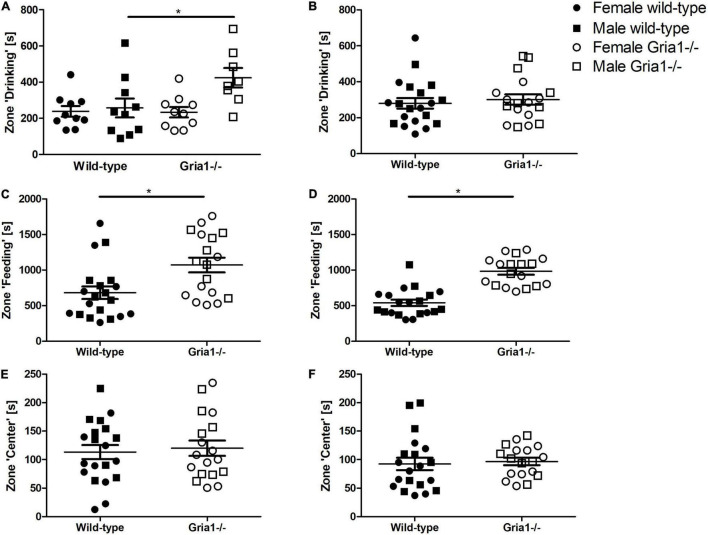
Bench-top assessment focusing on cage zones. We analyzed home-cage based activities per zone, assessing the duration the animals spent in predefined areas of the home cage. Concerning the zone “drinking,” there was a significant effect of genotype and a relevant genotype by sex interaction in prepubescent mice **(A)**, showing that male *Gria1^–/–^* mice spent more time in the zone “drinking” than age-matched male wild-types. In sexually mature mice **(B)**, we did not detect significant group differences for the time spent in the zone “drinking.” Prepubescent *Gria1^–/–^* mice spent significantly more time in the zone “feeding” than age-matched wild-types **(C)**. This was also the case for sexually mature *Gria1^–/–^* mice **(D)**. Considering the duration spent in the center of the cage, genotypes were statistically similar in prepubescent **(E)** and in sexually mature **(F)** mice. ANOVA, followed by FDR correction or Bonferroni multiple comparison *post hoc* tests. *n* = 8–10 per genotype per sex. *p < 0.05. Error bars indicate the standard error of the mean (SEM).

The analyses of the durations prepubescent mice spent in the zone “drinking” indicated a significant genotype by sex interaction (Figure 5A, ANOVA: genotype *F*_1,34_ = 3.75, *p* = 0.06, sex *F*_1,34_ = 6.27, *p* = 0.02, genotype by sex interaction *F*_1,34_ = 4.2, *p* = 0.048) with male *Gria1^–/–^* mice spending more time in the zone “drinking” than male wild-type littermates (Bonferroni *post hoc* test: *p* < 0.05). The analyses of durations in the zone “drinking” for sexually mature mice showed that genotypes and sexes were statistically similar (Figure 5B). Considering durations mice spent in the zone “feeding,” we detected a significant effect of genotype in prepubescent mice (Figure 5C, ANOVA: genotype *F*_1,34_ = 8.75, *p* = 0.006, FDR-adjusted *p* = 0.03) and in sexually mature mice (Figure 5D, ANOVA: genotype *F*_1,34_ = 45.59, *p* < 0.0001, FDR-adjusted *p* < 0.0001) indicating that *Gria1*^–/–^ mice spent significantly more time in the zone “feeding” than wild-type littermates. The time spent in the zone “center” did not significantly differ between genotypes and sexes in both prepubescent and sexually mature mice, and there were no relevant genotype by sex interactions (Figures 5E,F).

**FIGURE 6:**
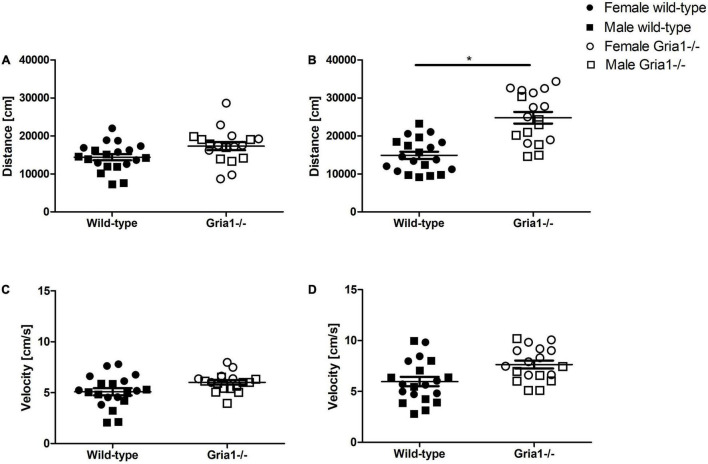
Bench-top assessment focusing on overall activity. We analyzed the overall activity focusing on the total distance moved and the velocity of movement in the PhenoTyper cages. The distance moved by prepubescent **(A)** and sexually mature **(B)** mice during the 60-min observation interval was significantly affected by genotype only in sexually mature mice. For the velocity of movement in prepubescent **(C)** and sexually mature **(D)** mice, a significant effect of genotype could not be confirmed. ANOVA, followed by FDR correction. *n* = 8–10 per genotype per sex. *p < 0.05. Error bars indicate the standard error of the mean (SEM).

Monitoring of the overall activity did not confirm a group difference in prepubescent mice (Figure 6A), but showed a significant effect of genotype in sexually mature mice (Figure 6B ANOVA: genotype *F*_1,34_ = 33.87, *p* < 0.0001, FDR-adjusted *p* < 0.0001) indicating that *Gria1^–/–^* mice moved a greater distance than wild-type littermates. However, the velocity of movement did not differ between genotypes in prepubescent and sexually mature mice (Figures 6C,D). Genotype effects of distance and velocity did not interact with sex in any measurement. Regardless of genotype, there was a sex difference in prepubescent mice indicating that females moved with higher speed than males (ANOVA: sex *F*_1,34_ = 13.32, *p* = 0.0009, FDR-adjusted *p* = 0.006). In contrast, data on distance and velocity from sexually mature mice did not confirm a significant effect of sex.

Considering social interaction displayed by the experimental units (two animals per cage), body contact durations were statistically similar between genotypes in prepubescent mice and in sexually mature mice (Supplementary Figure 2). Analyses of the mean distance between two subjects per cage showed a significant effect of genotype neither in prepubescent mice nor in sexually mature mice (Supplementary Figure 2). Sex differences and interactions were absent for all measurements of social interaction.

#### Open Field

**FIGURE 7:**
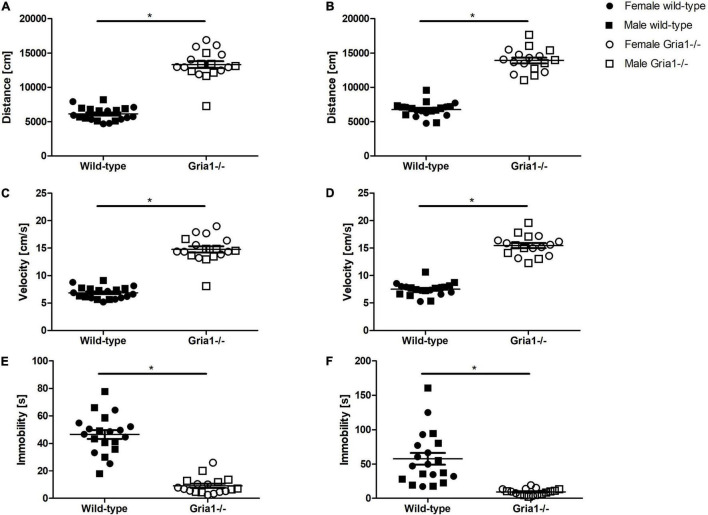
Open field test – total monitoring duration. During the total observation period of 15 min, *Gria1^–/–^* mice moved a greater distance than wild-type littermates during prepubescence **(A)** as well as once sexually mature **(B)**. Considering velocity of movement, *Gria1^–/–^* mice moved with increased speed as compared to age-matched wild-types during prepubescence **(C)** and once sexually mature **(D)**. Considering states of immobility displayed by the mice, there were significant effects of genotype demonstrating that *Gria1^–/–^* mice showed a shorter immobility duration than wild-type littermates during prepubescence **(E)** and once sexually mature **(F)**. ANOVA, followed by FDR-correction. *n* = 8–10 per genotype per sex. *p < 0.05. Error bars indicate the standard error of the mean (SEM).

Monitoring of the overall activity during the entire duration of 15 min revealed a significant effect of genotype in prepubescent and sexually mature mice. The analyses in prepubescent mice showed a significant effect of genotype on the total distance moved (Figure 7A, ANOVA: genotype *F*_1,34_ = 195.60, *p* < 0.0001; sex *F*_1,34_ = 1.77, *p* = 0.2; genotype by sex interaction *F*_1,34_ = 5.02, *p* = 0.03) and the velocity of movement (Figure 7C, ANOVA: genotype *F*_1,34_ = 191.60, *p* < 0.0001; sex *F*_1,34_ = 2.07, *p* = 0.2; genotype by sex interaction *F*_1,34_ = 4.645, *p* = 0.04): female and male *Gria1^–/–^* mice moved a greater distance (Bonferroni *post hoc* test: *p* < 0.0001 and *p* < 0.0001, respectively) and with increased velocity (Bonferroni *post hoc* test: *p* < 0.0001 and *p* < 0.0001, respectively) as compared to wild-type littermates. In sexually mature mice, analyses demonstrated a significant effect of genotype on distance (Figure 7B, ANOVA: genotype *F*_1,33_ = 232.5, *p* < 0.0001, FDR-adjusted *p* < 0.0001) and velocity (Figure 7D, ANOVA: genotype *F*_1,33_ = 232.40, *p* < 0.0001, FDR-adjusted *p* < 0.0001) indicating that *Gria1^–/–^* mice moved a greater distance and with increased velocity than wild-types. The total duration of immobility was significantly affected by genotype during prepubescence (Figure 7E, ANOVA: genotype *F*_1,34_ = 100.30, *p* < 0.0001, FDR-adjusted *p* < 0.0001) and sexual maturity (Figure 7F, ANOVA: genotype *F*_1,33_ = 25.43, *p* < 0.0001, FDR-adjusted *p* < 0.0001) reflecting that *Gria1*^–/–^ mice showed lower levels of immobility than wild-type littermates. Considering the duration animals spent in the zone “wall,” there was a significant effect of genotype interacting with sex in a significant manner observed in prepubescent mice (Supplementary Figure 3, ANOVA: genotype *F*_1,34_ = 16.98, *p* = 0.0002; genotype by sex interaction *F*_1,34_ = 5.161, *p* = 0.03), indicating that female *Gria1*^–/–^ mice showed higher levels of thigmotactic behavior than female wild-types (Bonferroni *post hoc* test: *p* < 0.0001). Analyses of the time mice spent in the zone “center” demonstrated a significant effect of genotype in prepubescent mice (Supplementary Figure 3, ANOVA: genotype *F*_1,34_ = 40.54, *p* < 0.0001, FDR-adjusted *p* < 0.0001) and in sexually mature mice (Supplementary Figure 3, ANOVA: genotype *F*_1,33_ = 13.21, *p* = 0.0009, FDR-adjusted *p* = 0.005) with lower “center” resting times in *Gria1^–/–^* mice than in wild-types, but this did not interact with sex, and there were no relevant sex effects for both measurements. Analysis of the frequency of the posture “rearing” revealed a significant effect of genotype only in sexually mature mice indicating that sexually mature *Gria1^–/–^* mice showed more rearing positions than wild-type littermates (Supplementary Figure 4, ANOVA: genotype *F*_1,34_ = 9.04, *p* = 0.0049, FDR-adjusted *p* = 0.03). Analyses of the frequency of “jumps” against the arena wall indicated a significant effect of genotype only in prepubescent mice with *Gria1^–/–^* mice showing fewer “jumps” than wild-type littermates (Supplementary Figure 4, ANOVA: genotype *F*_1,34_ = 10.18, *p* = 0.003, FDR-adjusted *p* = 0.02).

**FIGURE 8:**
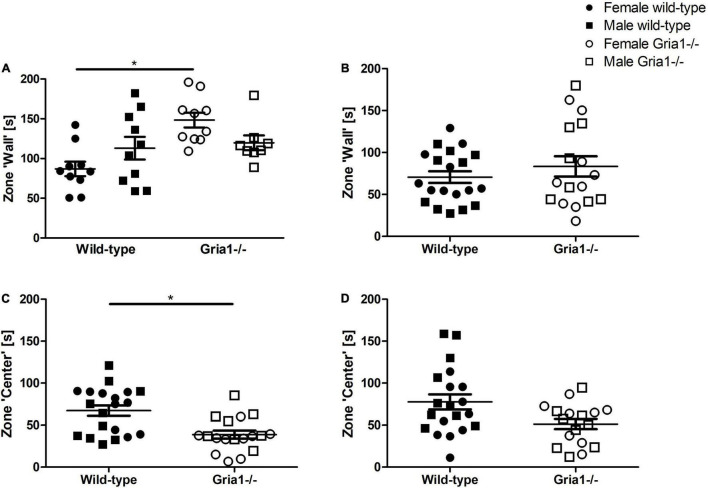
Open field test – monitoring of the first 5 min. Analysis of the first 5 min after exposure to the open field arena revealed that there was a significant effect of genotype and a significant genotype by sex interaction in prepubescent mice indicating that female *Gria1^–/–^* mice spent more time in the zone “wall” than female wild-type littermates **(A)**. In sexually mature mice, groups were statistically similar in the duration spent in the zone “wall” **(B)**. Considering the duration prepubescent **(C)** and sexually mature **(D)** mice spent in the zone “center” of the arena, there was a significant effect of genotype in prepubescent mice demonstrating that prepubescent *Gria1^–/–^* mice spent less time in the zone “center” than wild-type littermates. ANOVA, followed by FDR correction or Bonferroni multiple comparison *post hoc* tests. *n* = 8–10 per genotype per sex. *p < 0.05. Error bars indicate the standard error of the mean (SEM).

Measurement of activity during the first 5 min when exposed to the open field provides information about exploratory behavior. In prepubescent mice, analyses of the durations mice spent in the zone ‘wall’ showed a significant genotype by sex interaction (Figure 8A, ANOVA: genotype *F*_1,34_ = 9.72, *p* = 0.004, sex *F*_1,34_ = 0.014, *p* = 0.9, genotype by sex interaction *F*_1,34_ = 6.25, *p* = 0.02) indicating that female *Gria1*^–/–^ mice spent more time in the zone “wall” than female wild-types (Figure 8A, Bonferroni-*post hoc* test: *p* < 0.001). In sexually mature mice, the analysis of the time spent in the zone “wall” did not indicate significant group differences (Figure 8B). Analyses of the duration mice spent in the zone “center” revealed a significant effect of genotype only in prepubescent mice (Figure 8C, ANOVA: genotype *F*_1,34_ = 12.29, *p* = 0.001, FDR-adjusted *p* = 0.007) reflecting that *Gria1*^–/–^ mice spent less time in the zone “center” than wild-type littermates, while sex effects and interaction were not significant for both measurements. The durations sexually mature Gria1^–/–^ mice and wild-type mice spent in the zone “center” were statistically similar (Figure 8D).

##### Irwin Score

**FIGURE 9:**
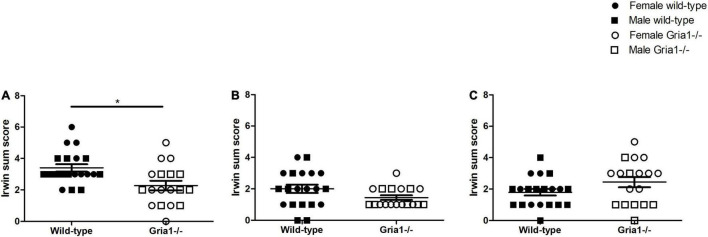
Irwin Score. The assessment of Irwin sum scores, calculated by adding up the score from all single parameters, revealed a significant group difference in prepubescent mice **(A)** with wild-type mice reaching higher sum scores than *Gria1^–/–^* mice. In pubescent mice **(B)** and sexually mature mice **(C)**, significant group differences were absent. ANOVA, followed by FDR correction or Bonferroni multiple comparison *post hoc* tests. *n* = 8–10 per genotype per sex. *p < 0.05. Error bars indicate the standard error of the mean (SEM). *p < 0.05. Error bars indicate the standard error of the mean (SEM).

The analyses of Irwin sum scores revealed a significant effect of genotype in prepubescent mice (Figure 9A, ART ANOVA: genotype *F*_1,34_ = 9.22, *p* = 0.005, FDR-adjusted *p* = 0.02): interestingly, prepubescent wild-types reached higher sum scores than age-matched *Gria1^–/–^* mice. Sum score analyses in pubescent mice indicated no group differences (Figure 9B). Sum scores in sexually mature mice were significantly affected by genotype and showed a significant genotype by sex interaction (Figure 9C, ART ANOVA: genotype *F*_1,34_ = 6.85, *p* = 0.013, sex *F*_1,34_ = 11.33, *p* = 0.002, genotype by sex interaction *F*_1,34_ = 6.89, *p* = 0.01). However, *post hoc* analyses failed to show a significant group difference in female and male mice (Bonferroni *post hoc* test *p* = 0.06 and *p* = 0.997, respectively). The analyses of single parameters revealed significant genotype effects of three handling-associated components: vocalization, urination, and defecation. Considering vocalization, there was a significant genotype effect in prepubescent mice (Supplementary Figure 5, ART ANOVA: *F*_1,34_ = 18.29, *p* = 0.0001, FDR-adjusted *p* = 0.001) indicating that wild-type mice reached higher scores than *Gria1^–/–^* littermates. Analyses of handling-associated urination scores showed a significant genotype effect in prepubescent mice (Supplementary Figure 5, ART ANOVA: genotype *F*_1,34_ = 12.54, *p* = 0.001, FDR-adjusted *p* = 0.007) showing that *Gria1^–/–^* mice had lower urination scores than wild-type littermates. Analysis of handling-associated defecation scores showed a significant genotype effect and a significant genotype by sex interaction in sexually mature mice (Supplementary Figure 5, ART ANOVA: genotype *F*_1,34_ = 4.41, *p* = 0.04, genotype by sex interaction *F*_1,34_ = 9.55, *p* = 0.004) indicating that female *Gria1^–/–^* mice had higher defecation scores than female wild-type littermates (Bonferroni *post hoc* test: *p* = 0.03). The analysis of body temperatures in prepubescent, pubescent and sexually mature mice failed to demonstrate significant group differences (Supplementary Figure 6).

##### Fecal Corticosterone Metabolites

**FIGURE 10:**
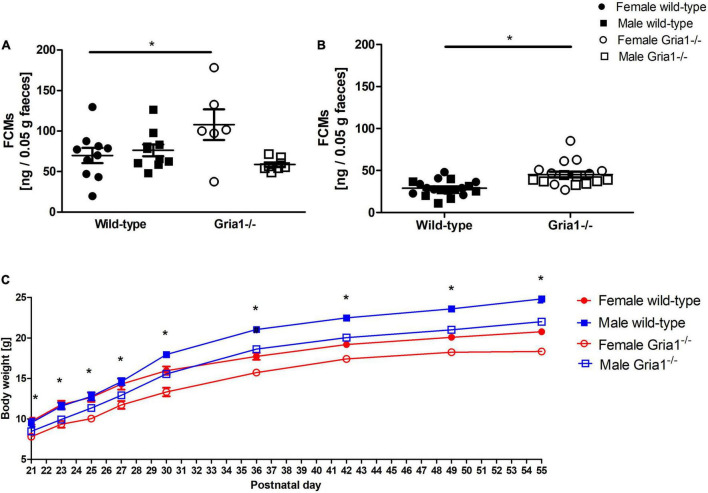
Fecal corticosterone metabolites (FCMs) and development of body weight. In prepubescent mice, there was a significant effect of genotype and a relevant interaction with sex, indicating higher metabolite levels in the female *Gria1^–/–^* group as compared to female wild-type littermates **(A)**. *Gria1^–/–^* mice exhibited higher metabolite levels than wild-type littermates once sexually mature **(B)**. Monitoring of body weight **(C)** confirmed a significant effect of genotype on all days of weight assessment with *Gria1^–/–^* mice reaching lower body weights than wild-type littermates. *n* = 6–10 per genotype per sex. ANOVA, followed by FDR correction or Bonferroni multiple comparison *post hoc* tests. *p < 0.05. Error bars indicate the standard error of the mean (SEM).

Analyses of fecal corticosterone metabolite (FCMs) showed a significant effect of sex and a significant genotype by sex interaction in prepubescent mice (Figure 10A, ANOVA: genotype *F*_1,30_ = 1.08, *p* = 0.3, sex *F*_1,30_ = 4.721, *p* = 0.04, genotype by sex interaction *F*_1,30_ = 7.929, *p* = 0.009) indicating that female *Gria1^–/–^* mice reached higher metabolite concentrations than female wild-type littermates (Bonferroni *post hoc* test: *p* < 0.05). Analyses of FCMs in sexually mature mice indicated a significant effect of genotype, while the main effect of sex and interaction with sex were both not significant (Figure 10B, ANOVA: genotype *F*_1,33_ = 8.34, *p* = 0.007, FDR-adjusted *p* = 0.03, sex *F*_1,33_ = 7.01, *p* = 0.01, FDR-adjusted *p* = 0.052, genotype by sex interaction *F*_1,33_ = 0.05, *p* = 0.8286): levels of FCMs were higher in *Gria1^–/–^* mice than in wild-type littermates.

##### Body Weight

Monitoring of body weight development revealed a significant effect of genotype on all 9 days of weight assessment: hereby, *Gria1^–/–^* mice showed reduced body weights when compared to wild-type littermates (Figure 10C, ANOVAs: P21 genotype *F*_1,34_ = 14.81, *p* = 0.0005, FDR-adjusted *p* = 0.003; P23 genotype *F*_1,34_ = 24.73, *p* < 0.0001, FDR-adjusted *p* < 0.0001; P25 genotype *F*_1,34_ = 20.2, *p* < 0.0001, FDR-adjusted *p* < 0.0001; P27 genotype *F*_1,34_ = 16.84, *p* < 0.0001, FDR-adjusted *p* < 0.0001; P30 genotype *F*_1,34_ = 32.9, *p* < 0.0001, FDR-adjusted *p* < 0.0001; P36 genotype *F*_1,34_ = 36.16, *p* < 0.0001, FDR-adjusted *p* < 0.0001; P42 genotype *F*_1,34_ = 38.75, *p* < 0.0001, FDR-adjusted *p* < 0.0001; P49 genotype *F*_1,34_ = 50.86, *p* < 0.0001, FDR-adjusted *p* < 0.0001; P55 genotype *F*_1,34_ = 66.35, *p* < 0.0001, FDR-adjusted *p* < 0.0001). Genotype by sex interactions did not become significant for any of the nine weight control days. Regardless of genotype, sex differences were detected from P30 onward with females reaching lower body weights than males (ANOVAs: P30 sex *F*_1,34_ = 23.09, *p* < 0.0001, FDR-adjusted *p* < 0.0001; P36 sex *F*_1,34_ = 71.29, *p* < 0.0001, FDR-adjusted *p* < 0.0001; P42 sex *F*_1,34_ = 76.82, *p* < 0.0001, FDR-adjusted *p* < 0.0001; P49 sex *F*_1,34_ = 102.5, *p* < 0.0001, FDR-adjusted *p* < 0.0001; P55 sex *F*_1,34_ = 143.1, *p* < 0.0001, FDR-adjusted *p* < 0.0001).

## Discussion

In line with our aim, we have identified behavioral and biochemical alterations in the *Gria1* knockout mouse model during the course of post-weaning development. Furthermore, we could demonstrate validity and feasibility of the newly designed composite measure scheme for the use in young mice of a loss-of-function genetic model of neuropsychiatric relevance. In this context, it is of importance that home-cage based approaches attracted increasing attention since they provide a non-intrusive, continuous, observer-independent, and, to a large extent, objective tool to uncover spontaneous activities (Pernold et al., 2019; Voikar and Gaburro, 2020). Investigations under standardized housing conditions reinforce reproducibility and external, inter-facility validity (Percie du Sert et al., 2020), and make practical implementation in the majority of conventional animal facilities possible. However, as applied here, the frequent presentation of novel objects in the home-cage, frequent handling of the young mice and the sequential testing situation represent confounding factors, which can essentially impact many of the readouts.

Among the parameters analyzed in the familiar environment of the mice, we examined the preference for saccharin, which we have previously introduced as a behavioral assay for severity assessment in laboratory rodents (e.g., Boldt et al., 2021). The innate preference for sweetness displayed by laboratory rodents can be considered to determine depression-related phenotypical traits (Crawley, 2006). The reduced preference for a sweet solution may indicate a reduced ability or inability to experience pleasure, which represents a key symptom of depression (Klein et al., 2015). Our previously generated baseline data from young wild-type mice (Reiber et al., 2022) suggest that the saccharin preference test can be applied in young mice from a prepubescent age onward. We have previously detected increased baseline preferences for saccharin in prepubescent and pubescent female mice, and a decline in sweetness preference becoming evident with reaching sexual maturity, which corresponds to sweetness preference in humans as reported in a scoping review by Venditti et al. (2020). In contrast, an earlier study conducted in rabbits reported a reduced sweetness preference displayed by younger animals (Reiber et al., 2022). Moreover, another more recent study on sucrose preference in young mice has successfully examined relevant strain differences in adolescent mice (Eltokhi et al., 2021). While there is no information available on sweetness preference in adolescent *Gria1^–/–^* mice, respective data from adult *Gria1^–/–^* mice draw an ambiguous picture so far: Austen and colleagues (Austen et al., 2017) reported that *Gria1^–/–^* mice aged 11–30 weeks showed reduced licking rates, but, in line with our findings in early- and late-adolescent deficient mice, normal sucrose consumption levels. Moreover, Strickland et al. (2021) suggest that GluA1 is not implicated in hedonic value, based on investigations in approximately 17- to 34-week-old mice. In young mice, higher intake of a sweet solution can also be considered as a mechanism to compensate energy loss or higher metabolic needs, especially in light of reduced post-weaning body weights indicating signs of developmental delay.

Toward examination of spontaneous activities, monitoring of burrowing activity and non-maternal nest building behavior have been introduced as approaches reflecting complex behaviors of laboratory rodents in their everyday environment (Deacon, 2006a,b). Since mice show high levels of intrinsic motivation for these “non-essential” activities, they can be viewed as a “positive indicator of well-being” (Jirkof, 2014). Inversely, observed aberrations may therefore indicate even slight signs of discomfort or a reduced ability to experience pleasure. Since burrowing and nesting both represent complex tasks, requiring persistent, goal-oriented, concentrated execution, they can provide a more in-depth understanding of normal behavioral function (Jirkof, 2014). Moreover, nest building has been suggested as a tool to evaluate sensory gating impairments in mice (Kraeuter et al., 2019). Sensory and motor gating deficits have been described as a clinically relevant phenotypical feature in adult *Gria1^–/–^* mice (Bannerman et al., 2004). A study analyzing nest building and burrowing performance in adult *Gria1* knockout mice revealed impaired nest building activity in male *Gria1^–/–^* mice as compared to male wild-type littermates, while burrowing performance was not affected by the *Gria1* genotype (Bannerman et al., 2004). In addition, Pedersen et al. (2014) have reported limited burrowing performance in an adult sub-chronic phencyclidine mouse model of schizophrenia.

Considering nest building activity in young mice, there are two relevant studies examining the ability of young mice to build nests which show diverging results: An earlier report from Moy et al. (2004) found relevant levels of nest building activity in mice aged 3–4 weeks, while Eltokhi et al. (2020) detected no relevant nesting behavior in adolescent mice. However, a range of interfering factors have to be considered when interpreting nesting data, among others, body weight, strain and housing conditions (Bult and Lynch, 1997; Martin et al., 2016; Robinson-Junker et al., 2017; Schwabe et al., 2020). Considering burrowing activity, earlier studies indicated relevant burrowing activity in adolescent mice (Hart et al., 2012; McLinden et al., 2012; Eltokhi et al., 2020). Here, we observed a significant relation between GluA1 deficiency and nest building performance in adolescent female and male mice. In addition, we found a reduction of overnight burrowing performance in sexually mature adolescent mice significantly related to the GluA1 deficiency. Limited nesting and burrowing performance observed in *Gria1*^–/–^ mice may be interpreted as a consequence of novelty-induced hyperactivity associated with the genetic deficiency (Sanderson et al., 2008; Aitta-Aho et al., 2019). In addition, recent evidence suggests increased activity of the hippocampus in GluA1 deficient mice (Bannerman et al., 2014) as a cause of poorer adaptation and impaired short-term memory (Taylor et al., 2011; Barkus et al., 2014; Aitta-Aho et al., 2019). When exposed to the new stimuli of nesting and burrowing material, hyperactive mice may face difficulties with paying attention to a specific task and targeted action.

Home-cage monitoring of activity provides information throughout the circadian rhythm (Pernold et al., 2019). Considering voluntary wheel running activity, an innate and self-rewarding behavior displayed by laboratory rodents, Häger et al. (2018) have demonstrated a high sensitivity for severity grading in mice. We have previously detected relevant levels of wheel running activity in young female and male wild-type mice from prepubescent age onward (Reiber et al., 2022). Considering wheel running in GluA1 deficient mice, there exist two studies analyzing the performance of adult *Gria1^–/–^* mice (Maksimovic et al., 2014; Ang et al., 2021). In line with our findings in adolescent *Gria1^–/–^* mice, Maksimovic et al. (2014) found no significant genotype-related difference during adulthood considering the distance run in the wheel. Ang et al. (2021) reported moderately reduced levels of overall wheel running activity in *Gria1^–/–^* mice with a significant activity reduction during the dark phase, suggesting relevant circadian rhythm impairments. Interestingly, a correlation between saccharin preference – which likewise represents a highly self-rewarding behavior – and voluntary wheel running activity has been described for wild-type mice, which reduced their intake of sucrose when simultaneously offered a freely accessible running wheel (Maksimovic et al., 2014).

Considering bench-top assessment, GluA1 deficient adult mice have been observed to show normal locomotor activity during continuous light/dark phase monitoring (Fitzgerald et al., 2010; Procaccini et al., 2011). However, as recently reported by Ang et al. (2021), *Gria1^–/–^* mice displayed significantly reduced locomotor activity in the dark phase and increased locomotor activity in the light phase as compared to the wild-type control group, indicating relevant circadian rhythm abnormalities related to the genetic deficiency. Thus, although hyperlocomotion and increased resting durations in the zone ‘feeding’ observed in adolescent *Gria1^–/–^* mice may be linked to lower post-weaning body weights, the activity levels measured are likely to be biased due to the selected short observation interval. In addition, relevant strain differences have been reported by Loos and colleagues (Loos et al., 2014), and there exists a number of external factors interfering with home-cage activity levels such as the presentation of novel objects and acclimatization to the cages.

When exposed to an open field arena in early and late adolescence, GluA1 deficient mice of both sexes displayed significant hyperlocomotion during both tests. This corresponds to earlier findings in adult *Gria1^–/–^* mice, which displayed pronounced hyperlocomotion after short-term exposure to a novel environment (Bannerman et al., 2004; Wiedholz et al., 2008; Fitzgerald et al., 2010; Procaccini et al., 2011; Aitta-Aho et al., 2019; Ang et al., 2021). After exposure to a novel home-cage environment, hyperlocomotion was found to decrease to baseline levels within 5–6 h (Procaccini et al., 2011). Notably, we observed pronounced thigmotaxis in female prepubescent *Gria1*^–/–^ mice. Thigmotaxis, in particular the tendency to remain close to the arena walls, was introduced by Simon et al. (1994) to grade levels of anxiety in mice. Since we applied a dimmed lighting level with low lux values during the open field test, increased durations in the wall zone can also point to deficits in goal-directed exploration of the entire arena.

Analysis from classical neurobehavioral scoring in mice as originally described by Irwin (1968) draws an ambiguous picture. Although alterations of handling-associated parameters were detected, we did not see an increase of sum scores associated with the genetic deficiency. Since Irwin scoring primarily provides methodological guidance on the standardized investigation of behavioral changes in preclinical drug assessment (Irwin, 1968; Moscardo et al., 2007; Lynch et al., 2011), it may show relatively poor results for severity grading in mouse lines burdened mild to moderately. Nevertheless, when repeatedly applied, Irwin scoring may provide a complementary tool to detect and track conspicuous changes of the autonomous nervous system over the developmental course.

The assessment of adrenocortical activity may depict slight signs of discomfort on a non-invasive basis in laboratory rodents (Touma et al., 2004; Palme, 2019). Nascent evidence from investigations in young wild-type mice suggests that determination of fecal corticosterone metabolites may provide sensitive information on preceding distress in laboratory mice during post-weaning development (Kolbe et al., 2015; Reiber et al., 2022). Increased levels of adrenocortical activity detected in prepubescent female *Gria1^–/–^* mice and in sexually mature *Gria1^–/–^* mice may indicate higher levels of distress experienced by the deficient mice in their home-cage environment. However, higher levels of FCMs can be biased because of higher metabolic needs, e.g., as a consequence of hyperlocomotion, and do not directly allow for conclusions about discomfort (Koolhaas et al., 2011). Regarding the first time point of sampling during prepubescence, it has to be considered that increased adrenocortical levels may be influenced by disturbing influences such as weaning and new group constellation as demonstrated by Kolbe et al. (2015).

Considering post-weaning weight assessment, we could demonstrate reduced body weights significantly related to the GluA1 deficiency. The reduction of body weight at certain postnatal days can represent signs of developmental delay. As stated by Talbot et al. (2020), body weight loss can provide a sensitive criterion in the context of severity assessment, once combined with further model-specific criteria in a behavioral approach.

In summary, we could demonstrate behavioral modifications during post-weaning development in mice with global GluA1 depletion. The behavioral readouts argue against a persistent or long-lasting level of distress in adolescent GluA1 deficient mice. The findings rather support mild temporarily-limited behavioral impairments, relevant to evidence-based welfare assessment. In particular, reduced burrowing behavior in late adolescence and reduced nesting performance in early and late adolescence indicate behavioral aberrations in the mice carrying the genetic deficiency, which can reflect a transient, mild level of burden. Activity patters, however, draw an ambiguous picture so far: while open field data point to hyperactivity and transient thigmotactic behavior, data from continuous home-cage based wheel running do not support an overall increase of activity. Moreover, activity patterns should be interpreted in light of the integrity of circadian rhythm, and confounding factors such as novelty of experimental setting and direct animal-observer-interaction should be carefully considered, this also applies to home-cage based testing batteries. In addition, sex-specific differences emphasize the relevance for the inclusion of both sexes for behavioral welfare-assessments in genetic mouse lines. Taken together, the transient, mild behavioral impairments support a classification of the overall harm burden of the line as “mild.” This laboratory-specific suggestion should be regarded as a recommendation, and animal husbandry-related factors should not be ignored. Moreover, further investigations in genetically modified mouse lines of neuropsychiatric relevance are necessary in order to confirm the robustness and generalizability of the candidate parameter selection.

## Data Availability Statement

The raw data supporting the conclusions of this article will be made available by the authors, without undue reservation. In addition, datasets are available on the DFG FOR2591 Online Repository. Available online at: https://for.severity-assessment.de/.

## Ethics Statement

The animal study was reviewed and approved by Government of Upper Bavaria, Maximilianstr. 39, 80538 Munich, Germany.

## Author Contributions

MR: study design, conduction of the experiments, data analysis and statistics, writing of the manuscript. HS: data analysis. RS: colony breeding and study design. PG: study design. RP: analysis of fecal corticosterone metabolites. HP: study concept and design, supervision, and writing of the manuscript. All authors: reviewed the manuscript.

## Conflict of Interest

The authors declare that the research was conducted in the absence of any commercial or financial relationships that could be construed as a potential conflict of interest.

## Publisher’s Note

All claims expressed in this article are solely those of the authors and do not necessarily represent those of their affiliated organizations, or those of the publisher, the editors and the reviewers. Any product that may be evaluated in this article, or claim that may be made by its manufacturer, is not guaranteed or endorsed by the publisher.

## Footnotes

1
https://www.basel-declaration.org

2
www.randomizer.org
