# Supplementary material for: Phenotyping Young GluA1 Deficient Mice – A Behavioral Characterization in a Genetic Loss-of-Function Model
Source: Front Behav Neurosci. 2022 Jun 2;16:877094. doi: 10.3389/fnbeh.2022.877094 (PMC9204703; doi:10.3389/fnbeh.2022.877094)
Supplement: Supplementary file 1 [file Data_Sheet_1.docx]

Supplementary Material

**Phenotyping young GluA1 deficient mice – a behavioral characterization in a genetic loss-of-function model**

Maria Reiber, Helen Stirling, Rolf Sprengel, Peter Gass, Rupert Palme and Heidrun Potschka

**^Supplementary Methods^**

**^Supplementary Figures S1-S6^**

**^Supplementary References^**

**^Supplementary Methods^**

*Nest building*

As described previously (Reiber et al., 2022), we applied the following scoring scheme for the image-based analysis of nest building performance: score 1 – nestlets are almost not manipulated (> 90% are intact); score 2 – nestlets are slightly manipulated (50-90% are intact); score 3 – nestlets are largely manipulated (50-90% are torn); score 4 – flat nest (> 90% are torn, shreds are placed in one quarter of the cage, < 50% of the walls are higher than the mouse); score 5 – nearly perfect nest (> 90% are torn, > 50% of the walls are higher than the mouse); score 6 – perfect nest (> 90% are torn, > 90% of the walls are higher than the mouse).

*Irwin Score*

As described previously (Reiber et al., 2022), we applied a slightly modified Irwin scale, divided into three consecutive parts:

1. Observation in the PhenoTyper home cages,

2. Observation during the open field test,

3. Observation and handling in fresh Makrolon type II open cages (Ehret GmbH & Co. KG, Emmendingen, Germany), enriched with bedding material (Lignocel Select, J. Rettenmaier & Söhne GmbH & Co. KG, Rosenberg, Germany).

Irwin scoring was carried out from the least invasive parameters assessed in the home cage to the more interfering, handling-associated parameters. An overview of the parameters is provided below.

Parameters assessed during observation in the PhenoTyper home cages:

*1) Body posture*

-2 flat, lying position, no muscle tone

-1 partly preserved muscle tone, occasional postural corrections

0 normal

+1 rigid, tense posture (e.g. opisthotonos)

+2 animals show no resting position, no analysis possible

*2) Position of pelvis*

-1 flattened

0 barely altered

+1 elevated

*3) Height of tail*

-1 tail repeatedly touching the ground during movement

0 normal tail position

+1 tail erected (max. 90 ° angle) during periods of resting and activity, possibly beating of tail

+2 tail erected (Straub-phenomenon)

*4) Limb rotation (outward)*

0 not present

+1 minimal

+2 clear

*5) Spontaneous locomotor activity*

-2 no activity

-1 reduced movement, occasional grooming

0 normal activity

+1 increased, possibly powerful, possibly angular movement

+2 restless motion type

*6) Ptosis*

0 eyelids open

+1 eyelid partly open

+2 eyelid closed

*7) Exophthalmoses*

0 no exophthalmoses

+1 minimal

+2 clear

*8) Lacrimation*

0 not present

+2 present

*9) Hypersalivation*

0 not present

+1 present

*10) Respiration*

+2 agonal respiration, acute respiratory distress

-1 reduced, irregular, tense respiration

0 normal respiration

+1 increased respiratory frequency

+2 increased respiratory rate is also present during resting periods

*11) Piloerection*

0 not present

+ 1 present: mild to moderate grade

+2 present: high grade

*12) Ataxia*

0 not present

+1 coordination problems

+2 loss of coordination

*13) Stereotypies*

0 not present

+2 present

*14) Freezing – towards presence of observer*

0 not present

+1 freezing slightly visible

+2 clear, abrupt freezing

*15) Trembling/tremor*

0 not present

+2 present

*16) Convulsions*

0 not present

+2 present

*17) Seizures*

0 not present

+2 present

*18) Vocalization*

0 not present

+1 occasional, spontaneous, quiet

+2 more frequent, spontaneous, louder

Parameters assessed during observation in the open field arenas:

*1) Ataxia*

0 not present

+1 coordination problems

+2 loss of coordination

*2) Freezing – when animal is placed into the arena*

0 not present

+1 freezing slightly visible

+2 clear, abrupt freezing

*3) Trembling/tremor*

0 not present

+2 present

*4) Convulsions*

0 not present

+2 present

*5) Seizures*

0 not present

+2 present

*6) Vocalization*

0 not present

+1 occasional, spontaneous, quiet

+2 more frequent, spontaneous, louder

Observation in fresh single cages and handling-associated parameters:

*1) Trembling/tremor*

0 not present

+2 present

*2) Convulsions*

0 not present

+2 present

*3) Seizures*

0 not present

+2 present

*4) Vocalization*

0 not present

+1 occasional, spontaneous, quiet

+2 more frequent, spontaneous, louder

*5) Curiosity towards a presented object (pen)*

-2 no reaction

-1 decreased curiosity

0 normal interest towards the presented object

+1 jerky pursuit, not adjusting to repeated withdrawal

+2 attack behavior

*6) Touch response*

-2 loss of response, no reaction even to increased stimuli

-1 repeated or increased stimulus provokes slow, reduced reaction (duck, evasion)

0 normal reaction to slight touching

+1 evasion of or ducking from slight touching

+2 flight over greater distance, attack behavior, vocalization to slight touching

*7) Startle response*

-2 no reaction

-1 delayed or decreased reaction

0 normal

+1 flight

+2 strong reaction, blepharospasm, laid-back ears, ducking on the ground or temporary freezing

*8) Irritability - bite propensity (handling)*

0 no irritability

+1 minimal

+2 clear

*9) Tone of body (handling)*

-2 completely limp

-1 reduced muscle tone

0 normal muscle tone

+1 increased muscle tone

+2 rigid

*10) Abdominal wall tension (handling)*

-1 reduced muscle tone

0 normal

+1 increased muscle tone

*11) Urination (handling)*

0 not present

+1 present

*12) Defecation (handling)*

0 not present

+1 present

*13) Feces*

-1 no or scarce amount of feces in the cage

0 character and amount of feces normal

+1 increased amount of feces

+2 almost liquid feces, diarrhea

*14) Positional reflexes*

Assessed only in case animal is continuously lying on the ground

-2 not present

-1 reduced

0 no alterations

*Analysis of fecal corticosterone metabolites*

As described previously (Reiber et al., 2022), the collection of fecal samples was carried out in the morning (07:00 am to 01:00 pm) directly after the open field paradigm. Feces of the mice were collected in the open field arenas, where the animals were placed and tested individually. In order to collect a sufficient amount of sample material, animals were then placed individually into Makrolon type II open cages (Ehret GmbH & Co. KG, Emmendingen, Germany), supplemented with bedding material (Lignocel Select, J. Rettenmaier & Söhne GmbH & Co. KG, Rosenberg, Germany), and feces were collected from these cages after two hours. The samples were stored frozen at -20°C. For processing, feces were dried and homogenized, and aliquots of 0.05 g were extracted with 1 ml of 80% methanol (Palme, Touma, Lepschy, Arias, & Dominchin, 2013). The samples were analyzed using a 5α-pregnane-3β,11β,21-triol-20-one enzyme immunoassay, which has been established and fully validated for the measurement of fecal corticosterone metabolites in mice (Touma, Palme, & Sachser, 2004; Touma, Sachser, Möstl, & Palme, 2003).

**^Supplementary Figures^**


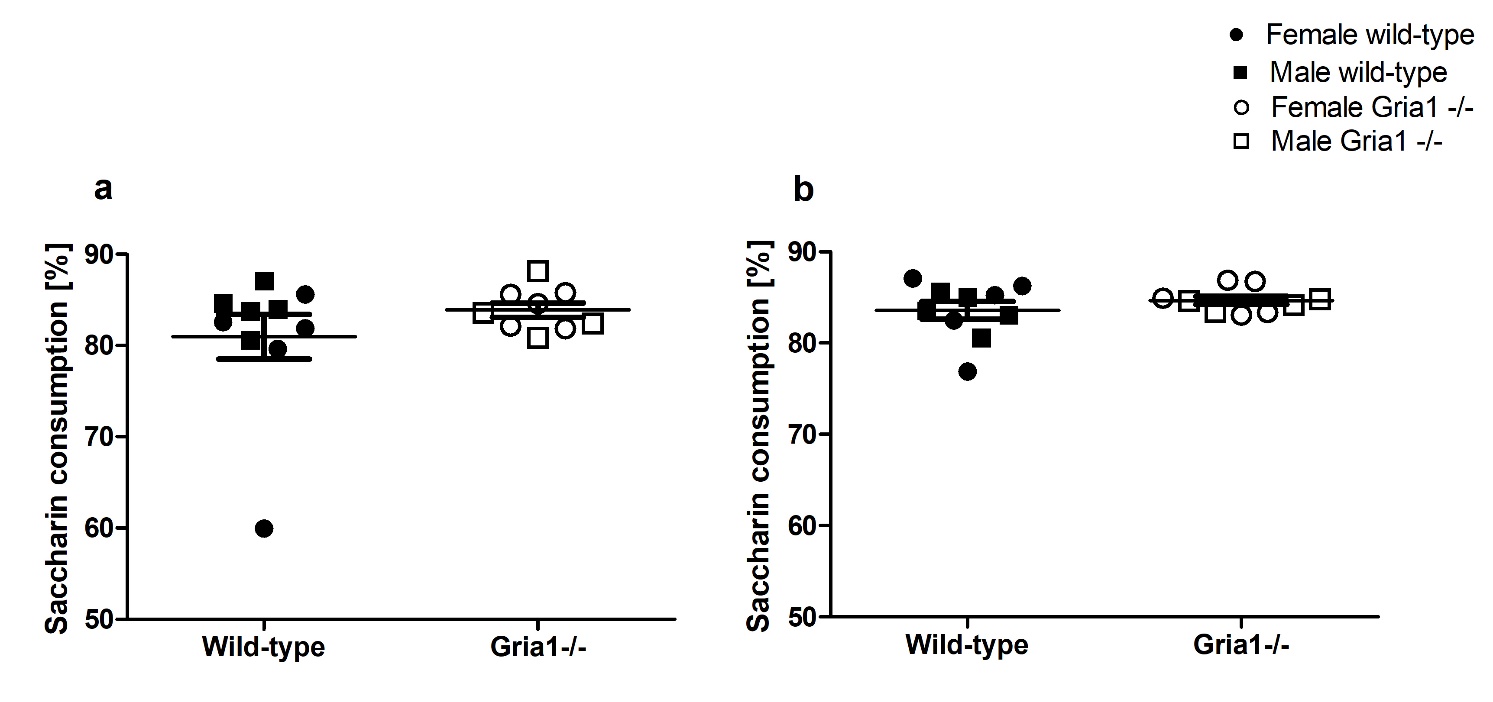


**Supplementary Figure S1.** Saccharin preference.

The preference for saccharin did not significantly differ between genotypes in prepubescent mice **(a)** and sexually mature mice **(b)**. Data from female and male mice were statistically similar and genotype by sex interactions were absent. ANOVA. *n*=4-5 per genotype per sex. * p<0.05. Error bars indicate the standard error of the mean (SEM).


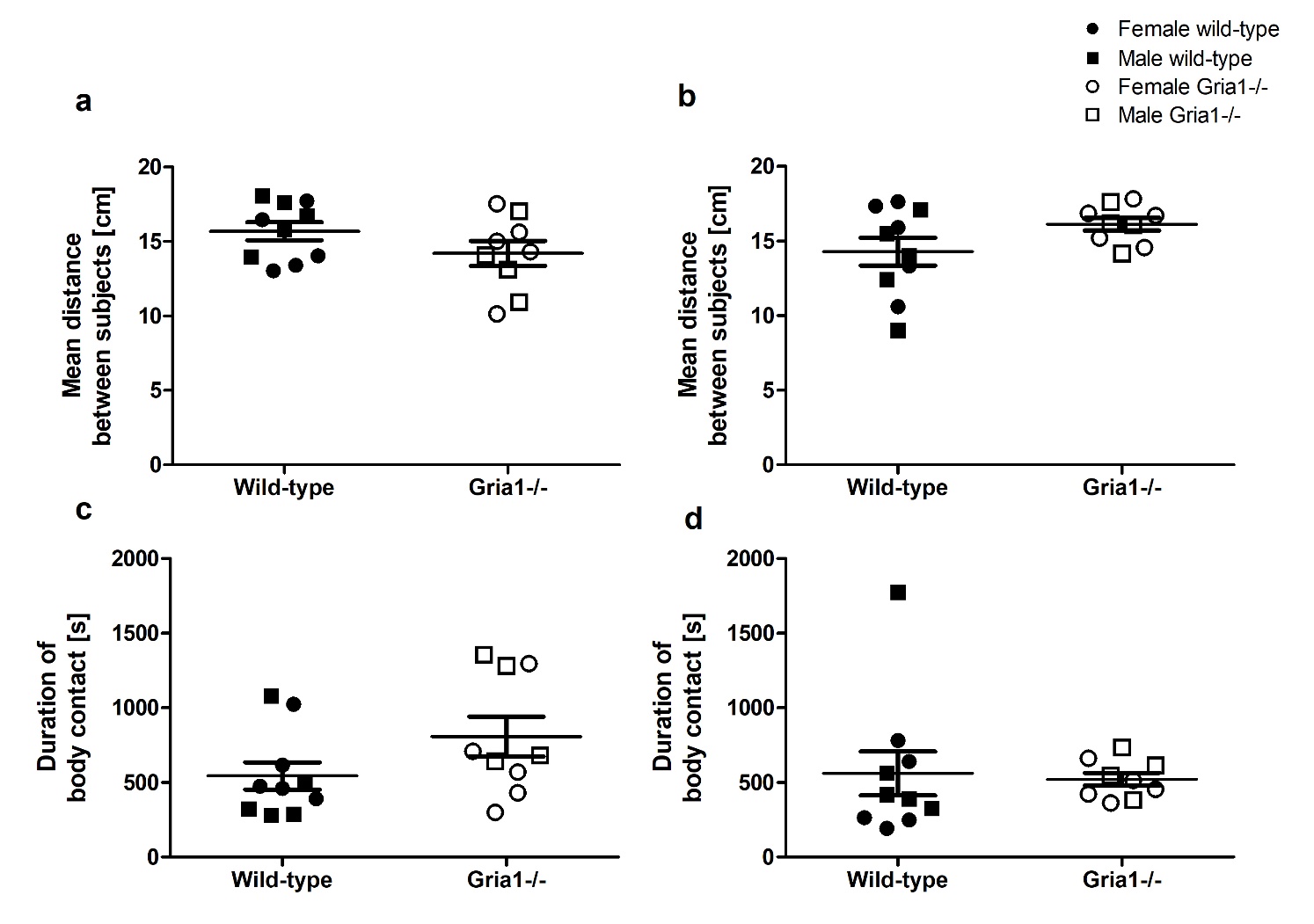


**Supplementary Figure S2.** Bench-top assessment of social interaction.

Social interaction parameters were analyzed per experimental unit focusing on the duration the mice spent in body contact and the mean distance between the two subjects per cage.

Analyses of the mean distance between two subjects per cage in prepubescent mice **(a)** and in sexually mature mice **(b)** indicated no significant effects of genotype. Body contact durations were statistically similar between genotypes in prepubescent mice **(c)** and in sexually mature mice **(d)**. Sex differences and genotype by sex interactions were absent for all measurements. ANOVA. *n*=4-5 per genotype per sex. * p<0.05. Error bars indicate the standard error of the mean (SEM).


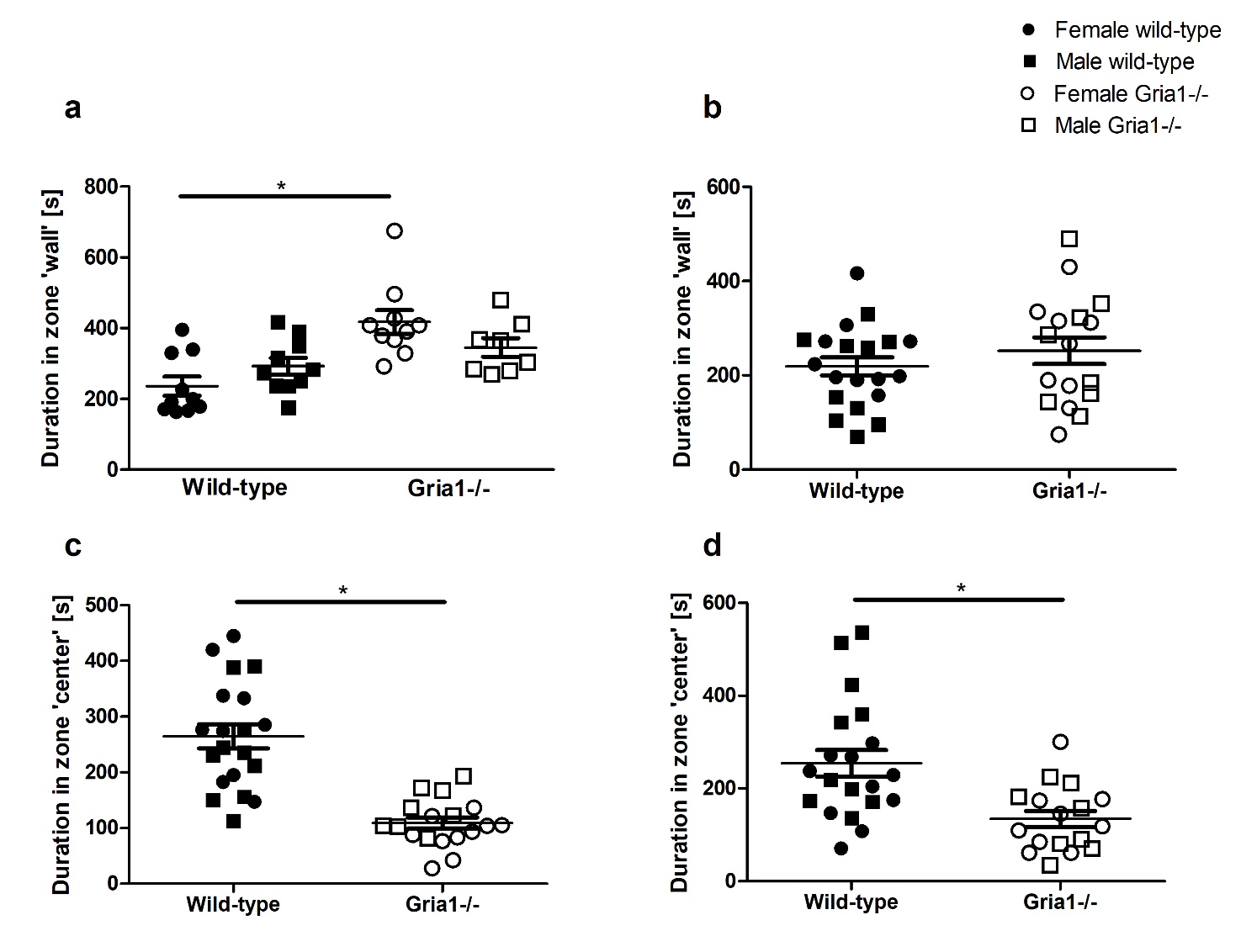


**Supplementary Figure S3.** Open field test – total monitoring duration.

The assessment of the time animals spent in the zones ‘wall’ and ‘center’ during the entire monitoring period of 15 minutes revealed significant genotype-related differences. Considering the duration mice spent in the zone ‘wall’ during prepubescence **(a)** and sexual maturity **(b)**, there was a significant effect of genotype and a significant genotype by sex interaction in prepubescent mice with female *Gria1*^-/-^ mice showing higher levels of thigmotactic behavior than female wild-types. Analyses of the time mice spent in the zone ‘center’ demonstrated a significant effect of genotype in prepubescent mice **(c)** and in sexually mature mice **(d)** with lower ‘center’ resting times in *Gria1^-/-^* mice than in wild-types, but this did not interact with sex, and there were no relevant sex effects. ANOVA, followed by FDR correction or Bonferroni multiple comparison post-hoc tests. *n*=8-10 per genotype per sex. * p<0.05. Error bars indicate the standard error of the mean (SEM).


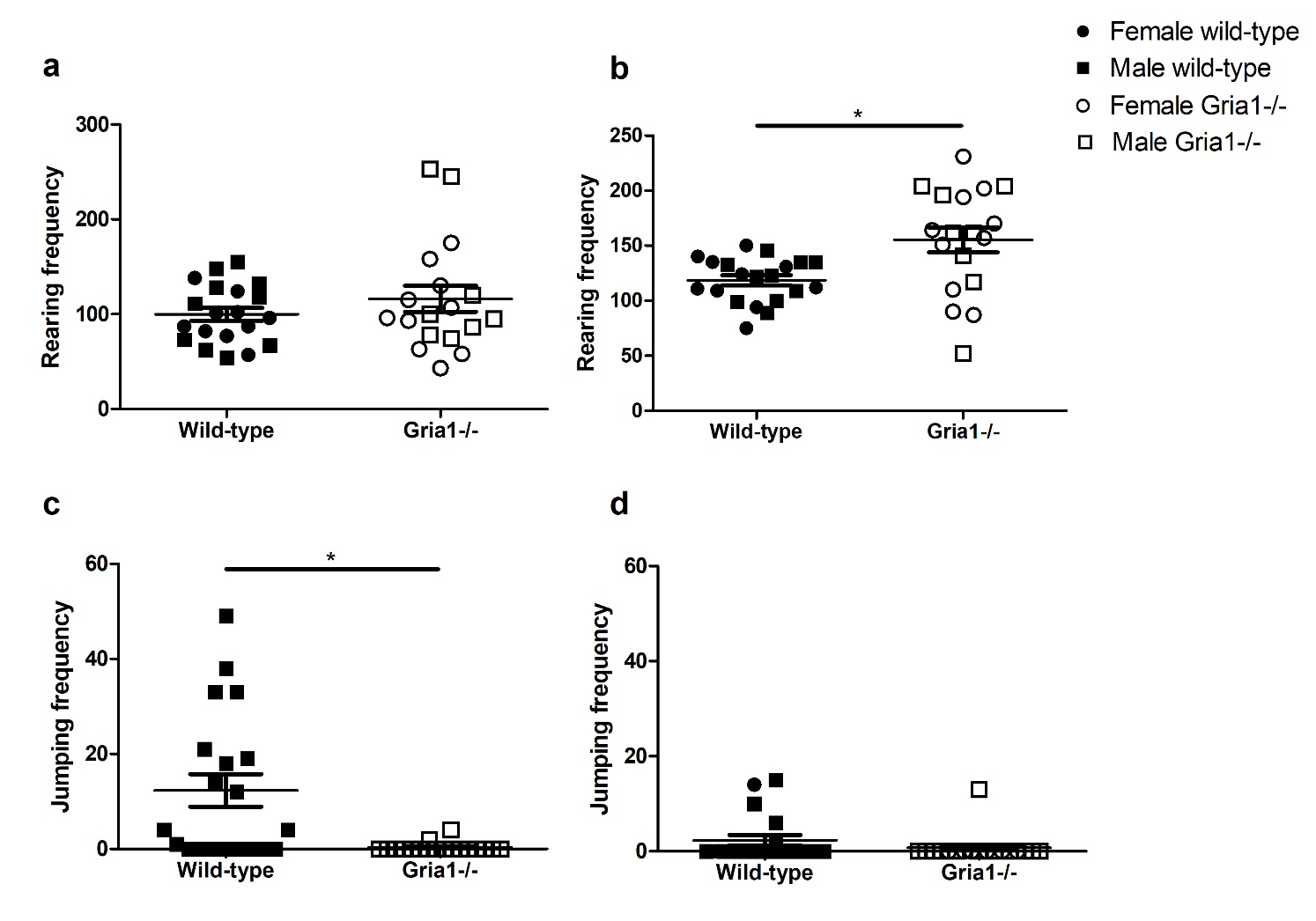


**Supplementary Figure S4.** Open field test – total monitoring duration.

Analysis of the frequency of the posture ‘rearing’ in prepubescent mice **(a)** and in sexually mature mice **(b)** revealed a significant effect of genotype only in sexually mature mice indicating that sexually mature *Gria1^-/-^* mice showed more rearing positions than wild-type littermates. Analyses of the frequency of ‘jumps’ against the arena wall in prepubescent mice **(c)** and sexually mature mice **(d)** indicated a significant effect of genotype only in prepubescent mice with prepubescent *Gria1^-/-^* mice showing fewer ‘jumps’ than wild-type littermates. ANOVA, followed by FDR correction. *n*=8-10 per genotype per sex. * p<0.05. Error bars indicate the standard error of the mean (SEM).


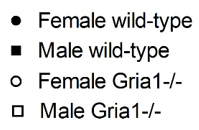

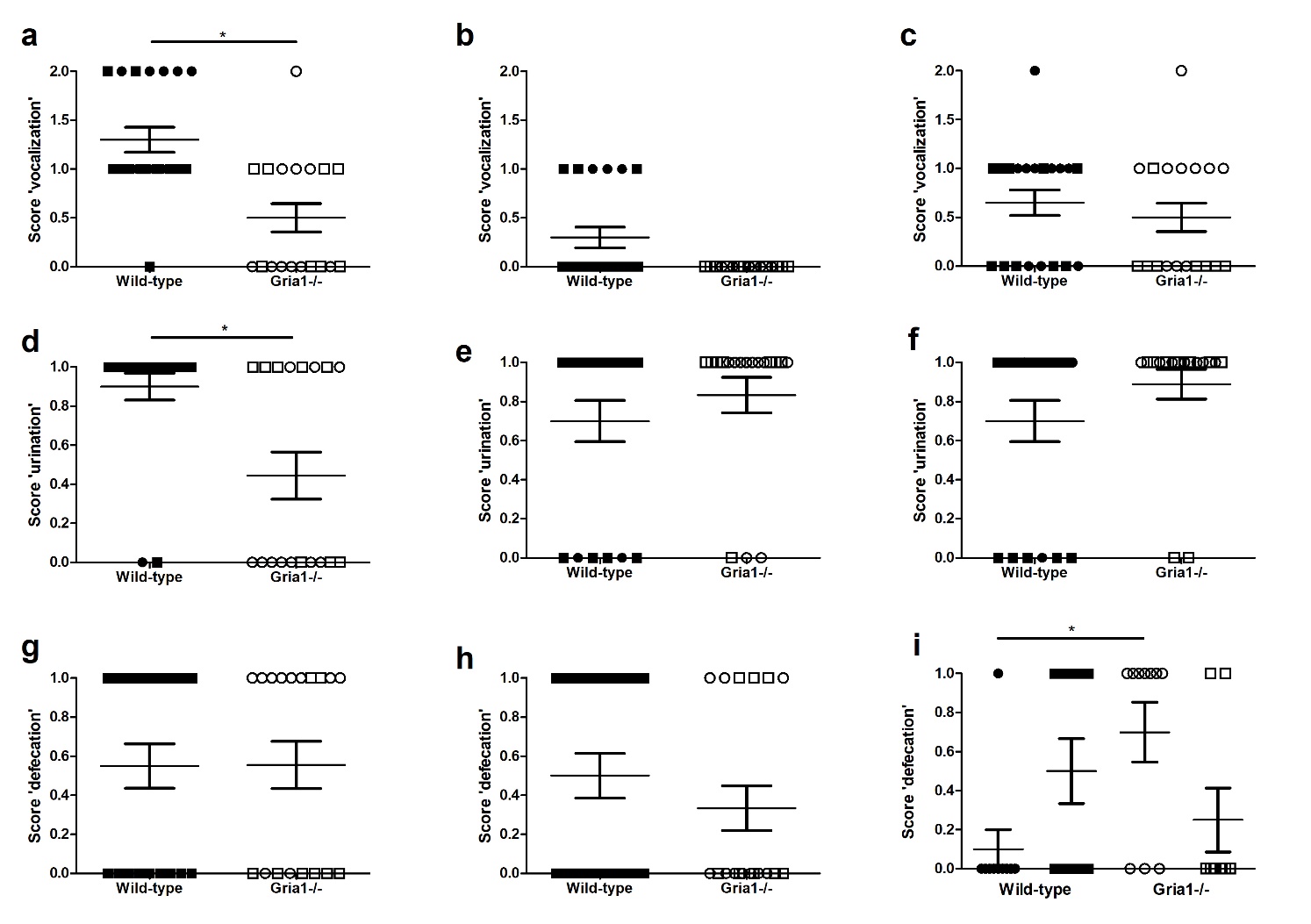


**Supplementary Figure S5.** Irwin Score.

The analysis of the Irwin scoring test revealed genotype-related group differences of handling-associated parameters. Considering vocalization assessed in prepubescent **(a)**, pubescent **(b)** and sexually mature **(c)** mice, there was a significant effect of genotype only in prepubescent mice indicating that wild-type mice reached higher vocalization scores than *Gria1^-/-^* littermates. Analyses of handling-associated urination scores in prepubescent **(d)**, pubescent **(e)** and sexually mature **(f)** mice showed a significant genotype effect in prepubescent mice. Analysis of handling-associated defecation scores during prepubescence **(g)**, pubescence **(h)** and sexual maturity **(i)** showed a significant genotype effect and a significant genotype by sex interaction in sexually mature mice indicating that female *Gria1^-/-^* mice reached higher defecation scores than female wild-type littermates. ART ANOVA, followed by FDR correction or Bonferroni multiple comparison post-hoc tests. *n*=8-10 per genotype per sex. * p<0.05. Error bars indicate the standard error of the mean (SEM).


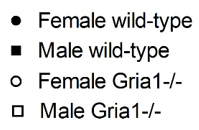


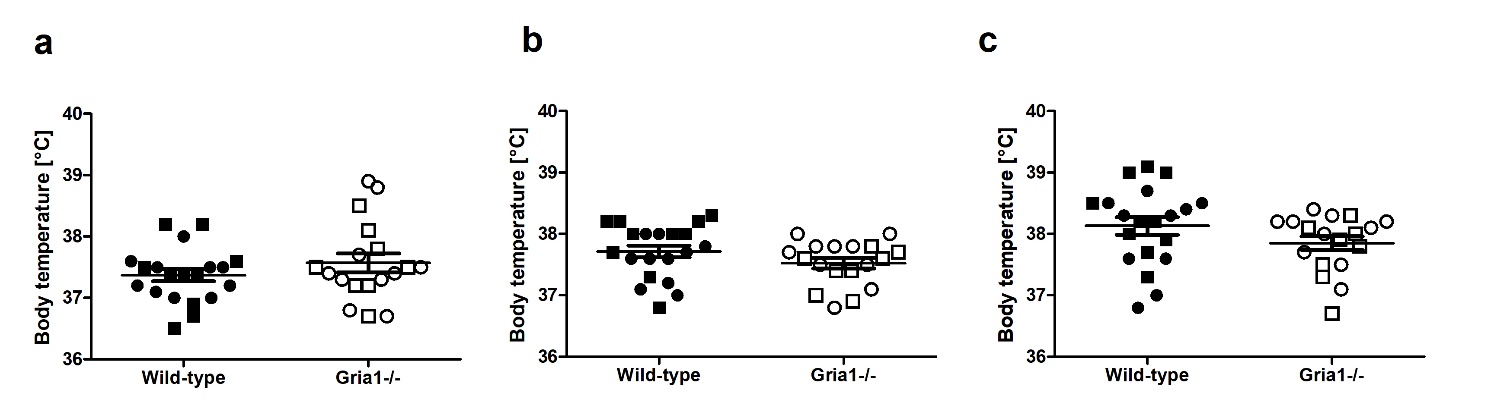


**Supplementary Figure S6.** Rectal body temperatures.

The measurement of body temperature showed no significant group differences between wildtype mice and *Gria1^-/-^* mice during prepubescence **(a)**, pubescence **(b)** and sexual maturity **(c)**. Sex differences were absent. ART ANOVA, followed by FDR correction. *n*=8-10 per genotype per sex. * p<0.05. Error bars indicate the standard error of the mean (SEM).

**^Supplementary References^**

Palme, Rupert, Touma, Chadi, Lepschy, M., Arias, N., & Dominchin, Florencia. (2013). Steroid extraction: Get the best out of faecal samples. *Wiener tierärztliche Monatsschrift, 100*, 238-246.

Reiber, M., Koska, I., Pace, C., Schönhoff, K., von Schumann, L., Palme, R., & Potschka, H. (2022). Development of behavioral patterns in young C57BL/6J mice: a home cage-based study. *Scientific Reports, 12*(1), 2550. doi: 10.1038/s41598-022-06395-1

Touma, C., Palme, R., & Sachser, N. (2004). Analyzing corticosterone metabolites in fecal samples of mice: a noninvasive technique to monitor stress hormones. *Horm Behav, 45*(1), 10-22. doi: 10.1016/j.yhbeh.2003.07.002

Touma, C., Sachser, N., Möstl, E., & Palme, R. (2003). Effects of sex and time of day on metabolism and excretion of corticosterone in urine and feces of mice. *Gen Comp Endocrinol, 130*(3), 267-278. doi: 10.1016/s0016-6480(02)00620-2
